# Behavioral addictions an updated look in the child and adolescent population

**DOI:** 10.1192/j.eurpsy.2025.943

**Published:** 2025-08-26

**Authors:** Y. D’Hiver Cantalejo, A. Helo Basha, V. Ros Fons, C. Delgado Gandia

**Affiliations:** 1 Psiquiatria, Hospital Nuestra Señora Del Prado, Talavera de la Reina, Spain

## Abstract

**Introduction:**

BEHAVIORAL ADDICTIONS AS A REPETITIVE PATTERN OF BEHAVIOR WITH DECREASE IN SELF-CONTROL AND A POWERFUL DESIRE TO CARRY OUT IT, DESPITE THE NEGATIVE CONSEQUENCES; WITH ACCOMPANYING SYMPTOMATOLOGY OF IRRITABILITY, CONCERN AND ANXIETY.

**Objectives:**

ANALYZE THE RESULTS ON REPORTS OF BEHAVIORAL ADICTIONS

**Methods:**

IN THE REPORTS ON BEHAVIORAL ADDICTIONS, ESTUDES (SURVEY ON THE DRUG USE IN SECONDARY EDUCATION IN SPAIN, 14-18 YEARS OLD) AND EDADES (SURVEY ON ALCOHOL AND DRUGS IN SPAIN) WE FOUND THE FOLLOWING:

- MANY MINORS PARTICIPATE IN GAMBLING GAMES.
- THE USE OF THE INTERNET, GAMBLING WITH MONEY AND VIDEO GAMES ARE VERY COMMON PRACTICES.
- ONLINE BETTING GAMES ARE MORE PREVALENT IN YOUNG PEOPLE.
- WITHIN THE PATHOLOGICAL GAME, A GREATER PREVALENCE OF BEHAVIOR IS OBSERVED RISK.
- WITHIN THE ABUSIVE USE OF THE INTERNET, GREATER CONSUMPTION OF CANNABIS AND ALCOHOL.

**Results:**

REGARDING THE TREATMENT, THE USEFULNESS OF COGNITIVE PSYCHOTHERAPY IS RECOGNIZED. BEHAVIORAL AND THE USE OF PSYCHODRUGS SUCH AS NALMEFENE AND NALTREXONE.

**Conclusions:**

ADDICTION TO THE INTERNET AND VIDEO GAMES COULD BE RECOGNIZED AS PSYCHIATRIC DIAGNOSES WHEN THEY LEAD MORE COMPLEX CHARACTERISTICS. SOME STUDIES PROPOSED TO TAKE INTO ACCOUNT THE EMOTIONAL NEEDS AND THE IMPULSIVITY, WHILE OTHERS, TALK ABOUT THE NEURO-BIOLOGICAL-GENETIC COMPONENT OF ADDICTIONS.

**Disclosure of Interest:**

None Declared

